# Role of Serotonin via 5-HT_2B_ Receptors in the Reinforcing Effects of MDMA in Mice

**DOI:** 10.1371/journal.pone.0007952

**Published:** 2009-11-23

**Authors:** Stéphane Doly, Jesus Bertran-Gonzalez, Jacques Callebert, Alexandra Bruneau, Sophie Marie Banas, Arnauld Belmer, Katia Boutourlinsky, Denis Hervé, Jean-Marie Launay, Luc Maroteaux

**Affiliations:** 1 INSERM U839, Paris, France; 2 Université Pierre et Marie Curie, Paris 6, Institut du Fer à Moulin, UMR-S0839, Paris, France; 3 AP-HP, Hôpital Lariboisière, Service de Biochimie, Paris, France; 4 INSERM U942, Paris, France; Chiba University Center for Forensic Mental Health, Japan

## Abstract

The amphetamine derivative 3,4-methylenedioxymethamphetamine (MDMA, ecstasy) reverses dopamine and serotonin transporters to produce efflux of dopamine and serotonin, respectively, in regions of the brain that have been implicated in reward. However, the role of serotonin/dopamine interactions in the behavioral effects of MDMA remains unclear. We previously showed that MDMA-induced locomotion, serotonin and dopamine release are 5-HT_2B_ receptor-dependent. The aim of the present study was to determine the contribution of serotonin and 5-HT_2B_ receptors to the reinforcing properties of MDMA.

We show here that 5-HT_2B_^−/−^ mice do not exhibit behavioral sensitization or conditioned place preference following MDMA (10 mg/kg) injections. In addition, MDMA-induced reinstatement of conditioned place preference after extinction and locomotor sensitization development are each abolished by a 5-HT_2B_ receptor antagonist (RS127445) in wild type mice. Accordingly, MDMA-induced dopamine D1 receptor-dependent phosphorylation of extracellular regulated kinase in nucleus accumbens is abolished in mice lacking functional 5-HT_2B_ receptors. Nevertheless, high doses (30 mg/kg) of MDMA induce dopamine-dependent but serotonin and 5-HT_2B_ receptor-independent behavioral effects.

These results underpin the importance of 5-HT_2B_ receptors in the reinforcing properties of MDMA and illustrate the importance of dose-dependent effects of MDMA on serotonin/dopamine interactions.

## Introduction

Activation of the mesolimbic dopaminergic system, which consists of projections from the midbrain ventral tegmental area (VTA) to forebrain regions, including the nucleus accumbens (NAcc), is critical for the psychostimulant and reinforcing effects of drugs of abuse [1]. Dopamine (DA) increase in the NAcc plays a critical role in reward and drug dependence and is a common response generated by all drugs of abuse [1]. On the other hand, emerging data support a role of serotonin (5-HT) in the rewarding effects of psychostimulants [2]. Serotonergic neurons from the dorsal raphé nucleus project to the VTA and the NAcc and impact dopaminergic neurotransmission [2], [3]. Thus, regulation of mesolimbic DA activity by 5-HT and its receptors plays an important role in the reinforcing effects of drugs of abuse [4], including the ‘club drug’ MDMA [5]–[8].

MDMA binds to and reverses the dopamine transporter (DAT) and the serotonin transporter (SERT) to produce carrier-mediated efflux of DA and 5-HT, respectively [9]. However, when access to SERT is blocked by selective serotonin reuptake inhibitors (SSRI), MDMA-evoked DA efflux in the NAcc is reduced [10]–[12]. In humans, relevant studies have shown that most of MDMA's effects are also markedly reduced after administration of 5-HT receptor antagonists or SSRIs, suggesting that these effects depend on SERT-mediated enhancement of 5-HT transmission [13]. In other words, MDMA-induced DA release in the NAcc is only partially carrier (DAT) - mediated but also involves a SERT-dependent 5-HT release.

Despite a widespread distribution in the central nervous system (CNS) [14]–[17], 5-HT_2B_ receptor function in the brain is mainly unknown. However, 5-HT_2B_ receptor mRNA and protein are coexpressed in SERT-expressing primary neurons from mice raphé nuclei [18]. This study showed that 5-HT_2B_ receptors govern the overall 5-HT transport system by promoting phosphorylation of SERT in these neurons [18]. Using reverse transcription polymerase chain reaction (RT-PCR), we recently confirmed that the 5-HT_2B_ receptor mRNA is expressed in mouse raphé nucleus [19], as previously observed in rats by DNA microarray and *in situ* hybridization [14]. Moreover, acute pharmacological inhibition or genetic ablation of the 5-HT_2B_ receptor in mice completely abolishes MDMA (10 mg/kg)-induced hyperlocomotion and 5-HT/DA release in NAcc and VTA [19]. Indeed, functional pre-synaptic 5-HT_2B_ receptors are required for MDMA-induced SERT dependent 5-HT release *in vivo* and *in vitro* [19]. The absence of 5-HT and DA release in the NAcc raises the question of the MDMA reinforcing effect in mice lacking functional 5-HT_2B_ receptors.

The main goal of the present study was to evaluate the role of 5-HT_2B_ receptors in activation of mesolimbic dopaminergic system and the reinforcing effects of MDMA. We first compared the behavioral effect of MDMA (10 and 30 mg/kg) in 5-HT_2B_^−/−^, RS127445 (selective 5-HT_2B_ receptor antagonist)-treated and WT (wild type) mice using locomotor sensitization. NAcc microdialysis studies were performed in awake WT, 5-HT_2B_^−/−^ and RS127445-treated WT mice to evaluate the effects of acute MDMA (30 mg/kg) injection on DA and 5-HT extracellular levels. We then performed conditioned place preference paradigms in WT, 5-HT_2B_^−/−^ and RS127445-treated WT mice to evaluate the role of 5-HT_2B_ receptor in the reinforcing effects effect of MDMA. Psychostimulants and other drugs of abuse, including MDMA, activate extracellular signal-regulated kinase (ERK) in the striatum, an essential component of a signaling pathway involved in synaptic plasticity and long-term effects of drugs of abuse. Thus, we quantified phosphorylated-ERK (p-ERK) immunoreactive neurons in the NAcc of WT and 5-HT_2B_^−/−^ mice after MDMA conditioning in the CPP paradigm. Finally, using *drd2*-EGFP transgenic mice, we characterized–for the first time– the populations of striatal dopaminergic neurons in which MDMA activates the pERK signaling pathways in mice expressing CPP.

## Materials and Methods

### Animals

5-HT_2B_^−/−^ mice used in these experiments were made in a pure 129Sv/PAS background. Wild type 129/SvPAS mice (8–10 week old) used as a control group were bred in-house. Swiss-Webster male mice (10 week-old) carrying *drd2*-EGFP bacterial artificial chromosome transgenes were generated by the Gene Expression Nervous System Atlas program at Rockefeller University (New York) [20]. Groups were composed of 50% male and 50% female for each experiment.

### Ethics Statement

Mice were kept under controlled environmental conditions (22°C, 12 h alternate light-dark cycles, 50% humidity, food and water *ad libitum*). The surgical procedures were performed under deep anesthesia (pentobarbital 25 µg/g, xylazine 20 µg/g). Behavioral tests and animal care were conducted in accordance with the standard ethical guidelines (National Institutes of Health's “Guide for the care and use of Laboratory animals”, and the European Communities Council European Communities Directive 86/609 EEC). All experiments involving mice were approved by the Ile de France Regional Ethics Committee for Animal Experiments.

### Microdialysis

Anesthetized animals were placed in a stereotaxic frame (D. Kopf, Tujunga, CA, USA) and a stainless-steel guide cannula (CMA/12, CMA Microdialysis, North Chelmsford, MA, USA; outer diameter 0.7 mm) was implanted in the NAcc. The cannula was then secured to the skull with dental cement, and the skin was sutured. Animals were kept in individual cages for a seven-day recovery. The microdialysis experiment was performed using awake mice. Dialysis probes were equipped with a Cuprophan membrane (membrane length 1 mm and diameter 0.24 mm, cutoff: 5,000 Da, Microdialysis AB, Sweden). According to Franklin and Praxinos (1997), stereotaxic coordinates in mm were for NAcc AP +1.2, ML +0.6, DV −4.2 both to bregma and dura surface, respectively. Probes were perfused at a constant rate of 5 µl/min with artificial CSF containing 154.1 mM Cl-, 147 mM Na+, 2.7 mM K+, 1 mM Mg2+, and 1.2 mM Ca2+, adjusted to pH 7.4 with 2 mM sodium phosphate buffer in awake animals. Dialysates were collected every 10 min. All measurements were carried out 150 min after the beginning of perfusion, by which time a steady state was achieved. Mice were injected with MDMA (30 mg/kg; i.p) 35 minutes after the beginning of measurements. Mice received a saline or RS127445 (0.5 mg/kg; i.p) injection 30 min before the MDMA injection. At the end of the experiment, all brains were fixed in a 4% formaldehyde solution and serial coronal slices were made on a microtome. Histological examination of cannula tip placement was subsequently made on 100 µm safranine-stained coronal sections. Dialysate samples were injected without any purification into an HPLC system that consists of a pump linked to an automatic injector (Agilent 1100, Palo Alto, CA, USA), a reverse-phase column (Zorbax SB C18, 3.5 lm, 150 · 4.6 mm; Agilent Technologies, Palo Alto, CA, USA) and a coulometric detector (Coulochem II; ESA Inc., Chelmsford, USA) with a 5011 analytical cell to quantify DA or 5-HT. The first electrode was fixed at -100 mV and the second electrode at +300 mV. The gain of the detector was set at 50 nA. The signal of the second electrode was connected to an HP Chemstation for HPLC. The composition of the mobile phase was 50 mM NaH_2_PO_4_, 0.1 mM Na_2_EDTA, 0.65 mM octyl sodium sulphate and 14% (v/v) methanol, pH 3.5. The flow rate was set at 1 ml/min.

### Locomotor Activity

Locomotor activity was measured in a circular corridor with four infrared beams placed at every 90° (Imetronic, France). Counts were incremented by consecutive interruption of two adjacent beams (i.e., mice moving through one-quarter of the corridor). Mice were injected with a saline solution and individually placed in the activity box for 30 min during 3 days consecutively for habituation before all locomotor experiments.

### Locomotor Sensitization

**Figure 1:**
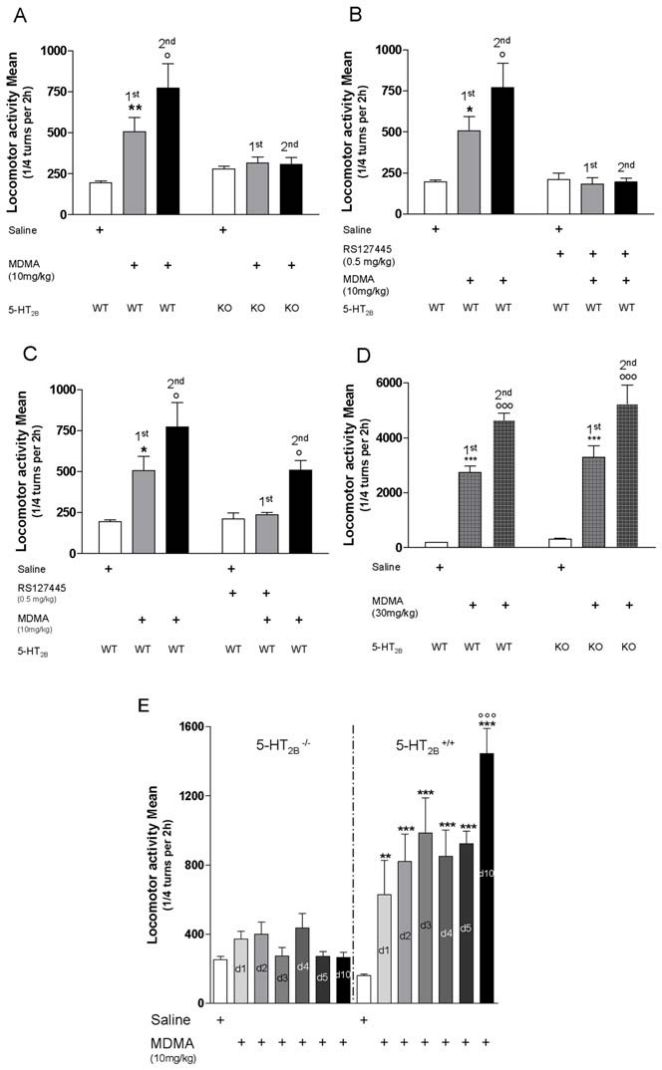
Effect of 5-HT2B receptor inhibition on locomotor activity and behavioral sensitization after MDMA injection. **Locomotor sensitization after MDMA two-injection protocol in WT and 5-HT_2B_^−/−^ mice (A–D):** MDMA (10 mg/kg i.p.) increases locomotor activity after the first injection (1^st^) in WT mice but not in 5-HT_2B_^−/−^ mice **A**) or RS127445-treated WT mice **B**). The stimulant effect of a challenge dose of MDMA (10 mg/kg) 7 days later (2^nd^) was significantly enhanced compared to the first injection in WT mice, while it had no effect in 5-HT_2B_^−/−^ mice **A**) or RS127445 pre-treated WT mice **B**). To evaluate the role of 5-HT_2B_ receptors in the development of locomotor sensitization, WT mice were treated with RS127445 30 min before the first injection of MDMA. The increased responsiveness to the challenge injection of MDMA in absence of RS127445 was totally abolished **C**). MDMA (30 mg/kg i.p.) induces locomotor activity after the first injection in WT and 5-HT_2B_^−/−^ mice compared to saline injection **D**). The stimulant effect of a challenge dose of MDMA (30 mg/kg) was significantly enhanced compared to the first injection in both WT and 5-HT_2B_^−/−^ mice **D**). Data (means±SEM, *n* = 8−14 per group) were analyzed by two-way ANOVA with genotype (**A–D**) or RS127445 pre-treatment (**B–C**) and MDMA treatment as main factors. A significant interaction was observed for the locomotor activity in figure **A**) *F* (2,66) = 12.86 *p*<0.01, and **B**) *F* (2,54) = 11.49 *p*<0.01, as well as a main effect of genotype *F* (2, 66) = 15.68, p<0.001 (**A**) or RS127445 pre-treatment *F* (2, 54) = 10.24, p<0.05 (**B**) and of MDMA treatment *F* (1,66) = 9.26, p<0.001 (**A**), and *F* (1,54) = 17.04, p<0.001 (**B**). No significant interaction was observed for the locomotor activity in figure **C**) *F* (2,66) = 3.57, ns, whereas a main effect of RS127445 pre-treatment at the 1^st^ MDMA injection, *F* (2,66) = 28.56, p<0.001, and of MDMA treatment *F* (1,66) = 6.64, p<0.01 were detected. Neither a significant interaction, *F* (2,50) = 0.25, ns, nor a main effect of genotype *F* (1,50) = 0.9, ns, was observed for the locomotor activity in figure **D**), whereas a main effect of MDMA treatment, *F* (2,50) = 82.72, *p*<0.001, was detected. Bonferroni tests were used for post-hoc comparisons. The null hypothesis was rejected at the p<0.05 level; *p<0.05; **p<0.01; ***p<0.001 compared to saline-treated mice. °p<0.05; °°°p<0.001 compared to MDMA 1^st^ injection. **Locomotor sensitization after repeated MDMA injection in WT and 5-HT_2B_^−/−^ mice (E):** MDMA (10 mg/kg i.p.) increases locomotor activity after the first injection (day 1; d1) in WT mice but not in 5-HT_2B_^−/−^ mice compare to saline injection. Repeated MDMA injection during the following days (Day 2 to 5, d2–d5) increases locomotor activity only in WT mice. The stimulant effect of a challenge dose of MDMA (10 mg/kg) 5 days later (day 10; d10) was significantly enhanced compared to the first injection in WT mice, while it had no effect in 5-HT_2B_^−/−^ mice. Data (means±SEM, *n* = 8 per group) were analyzed by two-way ANOVA with genotype and MDMA treatment as main factors. Bonferroni tests were used for post-hoc comparisons. The null hypothesis was rejected at the p<0.05 level; **p<0.01; ***p<0.001 compared to saline-treated mice. °°°p<0.001 compared to MDMA day 1 injection.

We used two models of locomotor sensitization in this study, a two-injection protocol [21] and a repeated drug injections protocol (Fig. 1). Mice received a first injection of MDMA and the locomotor activity was recorded for two hours (two-injection protocol). Mice were then challenged seven days later with a second injection of MDMA (test injection) and the locomotor activity was recorded for two hours. For testing the role of 5-HT_2B_ receptors in the induction of sensitization, mice received a saline or RS127445 (0.5 mg/kg) injection 30 min before the first MDMA injection. For the repeated drug injections protocol, mice received a daily injection of MDMA (10 mg/kg) during 5 days and the locomotor activity was recorded during two hours. Mice were then challenged 5 days later with a single injection (MDMA 10 mg/kg) and locomotor activity was recorded during two hours.

### CPP Acquisition

CPP experiment, consisting of three phases, was carried out following a procedure biased in terms of initial spontaneous preference [22]. CPP was assessed in a two-compartment apparatus (Imetronic, Pessac, France) with different patterns on floors and walls, separated by a central neutral area. During the first phase or pre-conditioning (Pre-C) mice were given access to both compartments of the apparatus for 30 min each day for four days. On day four, the time spent by the animal in each compartment was recorded for 30 min. In the second phase (conditioning), animals were conditioned with MDMA or saline through four pairings with the non-preferred or preferred compartment respectively. Mice received only one pairing each day. Animals conditioned with MDMA received an injection of MDMA 10 min before confinement in the drug-paired compartment for 30 min on days 5, 7, 9, and 11 and received physiological saline 10 min before being confined to the vehicle-paired compartment for 30 min on days 6, 8, 10, and 12; control animals received an injection of physiological saline 10 min before being confined for 30 min to each compartment alternatively. During the third phase or post-conditioning (Post-C), on day 13, the guillotine doors separating the two compartments were removed and the time spent by the mice (untreated) in each compartment was recorded during 30 min. The difference in seconds between the time spent in the drug-paired compartment in the Post-C test and that spent in the Pre-C test is a measure of the degree of conditioning induced by the drug (Score). If this difference is positive then the drug has induced a preference for the drug-paired compartment, whereas the opposite indicates the induction of an aversion.

### Extinction

Control and MDMA-conditioned groups underwent an extinction session during which the animals were placed in the apparatus (without guillotine doors separating the compartments) for 30 min until the time spent in the drug-paired compartment for each group conditioned with MDMA was similar to those of Pre-C (one extinction session per day during 12 days). MDMA-treated animals received the same number of extinction sessions, independently of their individual scores, as the criterion of extinction was a lack of significant difference with respect to Pre-C values. Saline conditioned groups only performed one extinction session to confirm the lack of CPP.

### Reinstatement

The effects of a priming dose of MDMA were evaluated 48 h after the confirmation of extinction. The tests of reinstatement were the same as for Post-C (free ambulation for 30 min) except that animals were tested 24 h after the administration of MDMA. For testing the role of 5-HT_2B_ receptors in the induction of reinstatement, mice received saline or RS127445 (0.5 mg/kg) 30 min before the MDMA priming injection.

### Tissue Preparation and Immunofluorescence

Twenty-four hours after CPP training, WT and 5-HT_2B_^−/−^ mice were re-exposed to saline, MDMA 10 mg/kg or MDMA 30 mg/kg. Ten minutes after re-exposure, mice were rapidly anesthetized with pentobarbital (30 mg/kg, i.p.; Sanofi-Aventis) and perfused transcardially with 4% (w/v) paraformaldehyde in 0.1 M sodium phosphate buffer, pH 7.5. Brains were postfixed overnight in the same solution and stored at 4°C. Thirty-micrometer-thick sections were cut with a Vibratome (Leica) and stored at –20°C in a solution containing 30% (v/v) ethylene glycol, 30% (v/v) glycerol, and 0.1 M sodium phosphate buffer, until they were processed for immunofluorescence. Brain regions were identified using a mouse brain atlas and sections equivalent to 1.54 mm from Bregma were taken. Sections were processed as previously described [23].

### Immunofluorescence Analysis

Confocal microscopy and image analysis were performed at the Institut du Fer à Moulin Imaging Facility. Double- and triple-labeled images from each region of interest were obtained bilaterally using sequential laser-scanning confocal microscopy (SP2; Leica). Neuronal quantification was performed in 375×375 µm images by counting nuclear EGFP fluorescence (for assessment of D2R-positive cells) and nuclear/cytoplasm Cy3 immunofluorescence (for each marker analyzed). Cell counts were performed by an observer unaware of the treatment received by the mice.

### Reagents

MDMA (Sigma-Aldrich, Saint-Quentin Fallavier, France) and 2-amino-4-(4-fluoronaphth-1-yl)-6-isopropylpyrimidine (RS127445) [24] (Tocris Bioscience, USA) were slowly dissolved in 0.9% (wt/vol) NaCl solution (saline). All drugs were administered i.p (0.1 ml/10 g body weight). RS-127445 was found to have sub-nanomolar affinity for the 5-HT_2B_ receptor (pK_i_ = 9.5±0.1) and at least 1,000 fold selectivity for this receptor as compared to numerous other receptors and monoamine uptake sites [24]. The acute pharmacological inhibition and genetic deletion of the 5-HT_2B_ receptor gives rise to an identical phenotype vis-à-vis MDMA-induced behavioral effect. Based on the initial study showing that RS127445 completely (1; 0.5 and 0.1 mg/kg; i.p) or partially (0.05 mg/kg) blocked MDMA-induced locomotion in WT mice and had no effect on basal locomotor activity [19] we used the 0.5 mg/kg dose (i.p).

### Statistics

Microdialysis data were analyzed by two-way ANOVA repeated measures with drug treatment and time as factors. Behavioral and biochemical assays were analyzed by two-way analysis of variance (ANOVA) with treatment and genotype as main factors. Bonferroni or Dunnett test were used for post hoc comparisons depending on the experiment. P<0.05 was predetermined as the threshold for statistical significance.

## Results

### Effect of 5-HT2B Receptor Inhibition on Behavioral Sensitization to Repeated MDMA Injection

Behavioral sensitization corresponds to a progressive enhancement of locomotor responses following repeated exposure to drug abuse [25]. Once established, sensitization is long-lasting since it is observed after re-exposure to the drug several weeks later [26], [27]. Locomotor sensitization is thought to underlie important aspects of vulnerability to drug addiction [28]. Since MDMA binds to DAT with a lower affinity than SERT [29] it may differentially affect these transporters, at low (10 mg/kg) or high (30 mg/kg) doses. We thus measured locomotor activity and behavioral sensitization in response to low (10 mg/kg) and high doses (30 mg/kg) of MDMA in WT, 5-HT_2B_^−/−^ or RS127445-treated wildtype mice (Figure 1). Figure 1A shows that acute injection (1^st^ injection) of MDMA (10 mg/kg) increases locomotor activity compared to saline injection in WT mice. The stimulant effect of a challenge dose of MDMA (10 mg/kg) seven days later (2^nd^ injection) was significantly enhanced compared to the first injection in WT mice. However, neither the first nor the second injection induced locomotion in 5-HT_2B_^−/−^ mice (Figure 1A) or RS127445-treated mice (Figure 1B) compared to saline injection. In the same way, locomotor sensitization induced with a repeated injection paradigm (i.e. daily injection of MDMA 10 mg/kg during 5 days) was also abolished in 5-HT_2B_^−/−^ compared to WT mice (Fig. 1E).

To evaluate the role of 5-HT_2B_ receptors in the locomotor sensitization induction, WT mice were treated with a selective 5-HT_2B_ receptor antagonist (RS127445; 0.5 mg/kg) 30 min before the first injection of MDMA. The challenge injection of MDMA was then performed in absence of RS127445; the increased responsiveness to the challenge injection (2^nd^) of MDMA was thus totally abolished, supporting a role for the 5-HT_2B_ receptor in the induction of sensitization (Figure 1C).

By contrast, in both WT and 5-HT_2B_^−/−^ mice, a high dose of MDMA (30 mg/kg) induced a 30-fold increase in locomotion and a significant increase in locomotor sensitization (Figure 1D). These experiments indicate that, unlike low doses, high doses of MDMA induce a 5-HT_2B_ receptor-independent sensitization in mice.

### Effect of 5-HT_2B_ Receptor Inhibition on MDMA (30 mg/kg)-Evoked Increase in 5-HT and DA Level in NAcc as Measured by *In Vivo* Microdialysis

**Figure 2:**
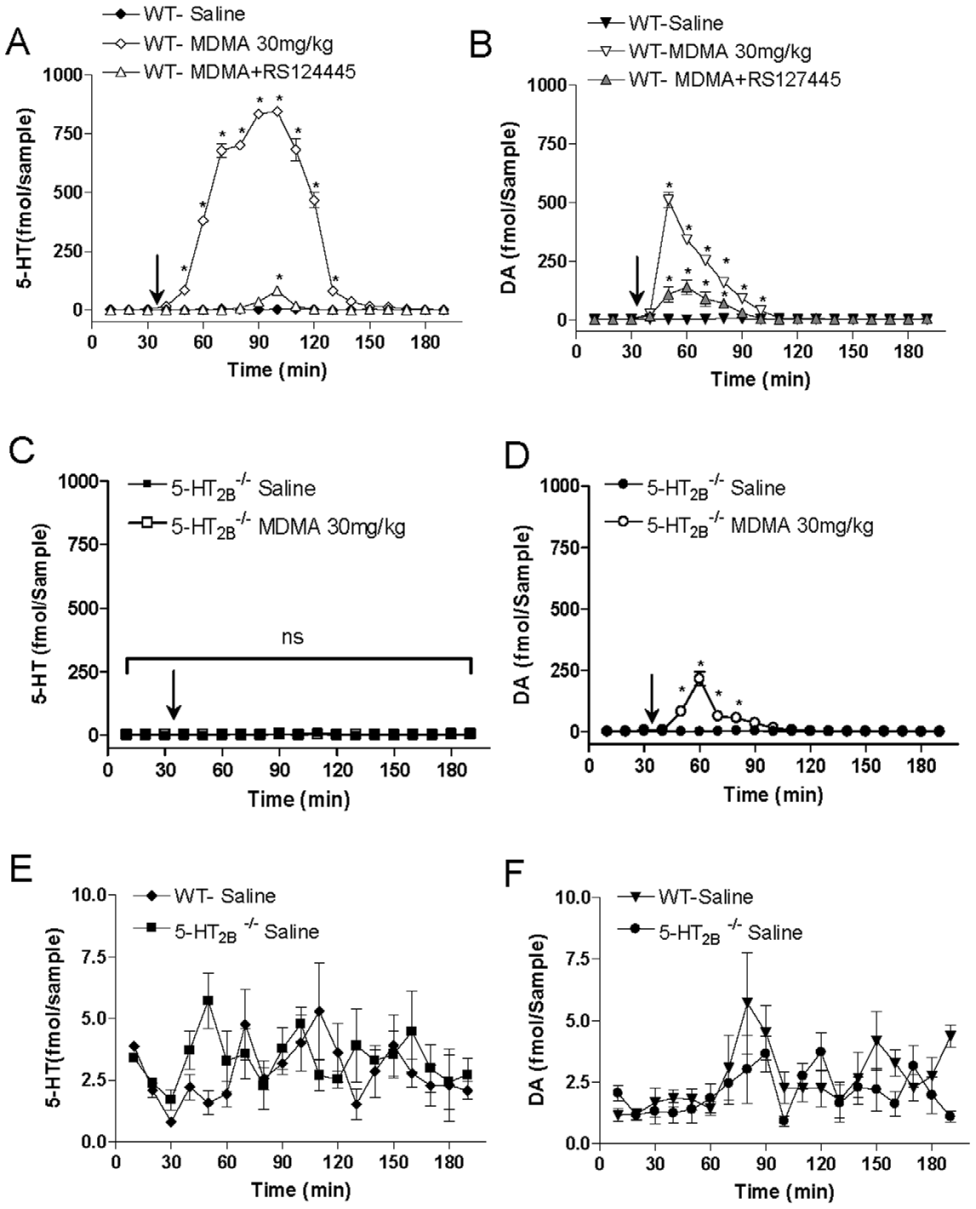
Effect of 5-HT2B receptor inhibition on MDMA (30 mg/kg) - evoked increase in NAcc 5-HT and DA levels as measured by *in vivo* microdialysis in awake mice. Effect of MDMA (30 mg/kg i.p.) or saline injection on (**A**) 5-HT, (**B**) DA concentrations in dialysates from the NAcc of WT or RS127445 (0.5 mg/kg i.p.)-WT pre-treated mice. Effect of 5-HT2B receptor genetic ablation on MDMA (30 mg/kg)-induced changes in (**C**) 5-HT or (**D**) DA levels in NAcc. MDMA or saline solutions were injected 35 minutes after test began (arrow). Basal 5-HT and DA extracellular levels are reported at lower scale in **E**) and **F**) respectively for WT and 5-HT2B-/- mice. Data (means±SEM, *n* = 4−5 per group) were analyzed by two-way ANOVA (repeated measures) with RS-administration or genotype and time as main factors. A significant interaction was observed after 5-HT2B receptor pharmacological inhibition (RS127445) (**A**–**B**) for 5-HT levels *F* (36,180) = 429.5 *p*<0.001 as well as for DA levels *F* (36,180) = 82.43, *p*<0.001. A main effect of RS-treatment was observed for 5-HT levels *F* (2, 180) = 3677, p<0.001 and for DA levels *F* (2, 180) = 449.7, p<0.001, whereas a main effect of time was also detected for 5-HT levels *F* (18, 180) = 498.1, p<0.001 and for DA levels *F* (18, 180) = 166.5, p<0.001. For 5-HT2B-/- mice (**C**–**D**), a significant interaction was not observed for 5-HT levels *F* (18,126) = 1.33, ns, but it was the case for DA levels *F* (18,126) = 53.98, *p*<0.001. Neither a main effect of genotype *F* (1, 126) = 0.36, ns, nor of time *F* (18, 126) = 1.08, ns, was observed for 5-HT levels. On the contrary, a main effect of genotype *F* (1, 126) = 363.3, p<0.001, as well as of time *F* (18, 126) = 53.55, p<0.001 was detected for DA levels. Bonferroni tests were used for post-hoc comparisons. The null hypothesis was rejected at the p<0.05 level; *p<0.001.

We have previously shown that MDMA (10 mg/kg)-induced hyperlocomotion, 5-HT and DA release are abolish in 5-HT_2B_^−/−^ or RS127445 treated mice [19]. In order to understand the contrasting behavioral results observed between low (10 mg/kg) and high (30 mg/kg) doses of MDMA, we compared changes in accumbal 5-HT and DA extracellular concentrations in awake WT, 5-HT_2B_^−/−^ and RS127445-treated mice (Figure 2). In WT mice, MDMA (30 mg/kg) induced a 160-fold increase in extracellular 5-HT levels in the NAcc within 80 minutes (Figure 2A), an effect that was absent in RS127445-treated or 5-HT_2B_^−/−^ mice (Figure 2A and 2C respectively). However, MDMA (30 mg/kg) caused a 100-fold increase in extracellular DA concentration in the NAcc of wild type mice within 50 minutes (Figure 2B); in 5-HT_2B_^−/−^ or RS127445-treated mice, MDMA elicited a 50-fold increase in synaptic DA levels in the NAcc within 30 minutes (Figure 2B and 2D respectively). Basal 5-HT and DA extracellular level are reported in Figure 2E and 2F respectively for WT and 5-HT_2B_^−/−^ mice. Therefore, the dependence of MDMA-induced 5-HT release on 5-HT_2B_ receptors persists even at a high dose (30 mg/kg) of MDMA; the remaining MDMA-induced DA release, however, appears to be partially independent of 5-HT_2B_ receptors and may explain the hyperlocomotion and locomotor sensitization seen in 5-HT_2B_^−/−^ mice that are not observed at 10 mg/kg of MDMA.

### Effect of 5-HT_2B_ Receptor Inhibition on MDMA-Induced CPP and CPP Reinstatement

**Figure 3:**
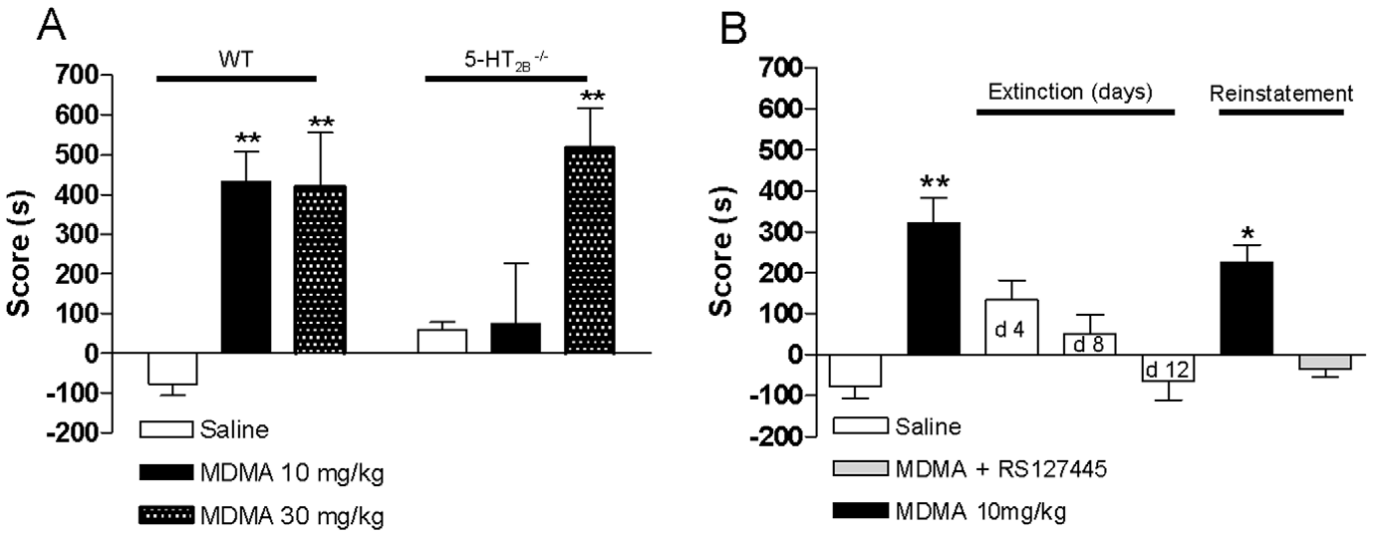
Effect of 5-HT2B receptor inhibition on MDMA-induced conditioned place preference in mice. **A**) Repeated i.p. injection of MDMA 10 mg/kg induced CPP in WT mice compared to saline injection, but this effect was absent in 5-HT_2B_^−/−^ mice. However, repeated injection of a high dose of MDMA (30 mg/kg) induced CPP in WT as well as in 5-HT_2B_^−/−^ mice. Data (means±SEM, *n* = 8 per group) were analyzed by two-way ANOVA with genotype and treatment as main factors, revealing a significant interaction, *F*(2, 41) = 3.93, p<0.05, a main effect of treatment *F*(2, 41) = 11.67, p<0,001 but no effect of genotype *F*(1, 41) = 0.27, ns. Bonferroni tests were used for post-hoc comparisons. In all cases, p<0.05 was considered statistically significant; **p<0.01 compared to saline-treated mice. **B**) After an initial extinction of the CPP (12 days) in WT mice, MDMA (10 mg/kg) re-exposure induced reinstatement of CPP. MDMA-induced reinstatement was not observed in RS127445-pretreated mice. Data (means±SEM, *n* = 10−20 per group) were analyzed by one-way ANOVA. Dunnetts tests were used for post-hoc comparisons. The null hypothesis was rejected at the p<0.05 level; *p<0.05 **p<0.01 compared to saline-treated mice.

In order to evaluate the role of 5-HT_2B_ receptors in the reinforcing effects of MDMA, we compared WT and 5-HT_2B_^−/−^ mice in the CPP paradigm. CPP is a robust model used to assess the addictive properties of drugs of abuse [22]. This procedure is based on the fact that the pairing of neutral distinctive environmental stimuli with a drug results in an acquired preference for those specific stimuli. As shown in Figure 3A, repeated injection of MDMA (10 mg/kg) in the paired compartment induces CPP in WT mice compared to saline-injected WT mice. In contrast, MDMA (10 mg/kg)-induced CPP in WT mice was not observed in 5-HT_2B_^−/−^ mice. However, as observed for locomotor sensitization, a high dose of MDMA (30 mg/kg) did induce CPP in both WT and 5-HT_2B_^−/−^ mice (Figure 3A).

Reinstatement of drug-seeking behavior in animals is relevant to drug relapse in humans. We employed the CPP paradigm to investigate the extinction and reinstatement of the place-conditioned response to MDMA injection, a model that is consistent with drug-seeking behavior. Figure 2B shows that MDMA (10 mg/kg) re-exposure induces reinstatement of CPP after an initial extinction of CPP (12 days) in WT mice. To evaluate the role of the 5-HT_2B_ receptor in the reinstatement of CPP, mice expressing CPP extinction were treated with a selective 5-HT_2B_ receptor antagonist (RS127445, 0.5 mg/kg ip) or saline solution 30 min before MDMA re-exposure. MDMA-induced reinstatement was completely blocked in RS127445-pretreated mice, indicating that 5-HT_2B_ receptors are required for the induction of CPP reinstatement.

### Effect of 5-HT2B Receptor Inhibition on MDMA-Induced ERK Activation

**Figure 4:**
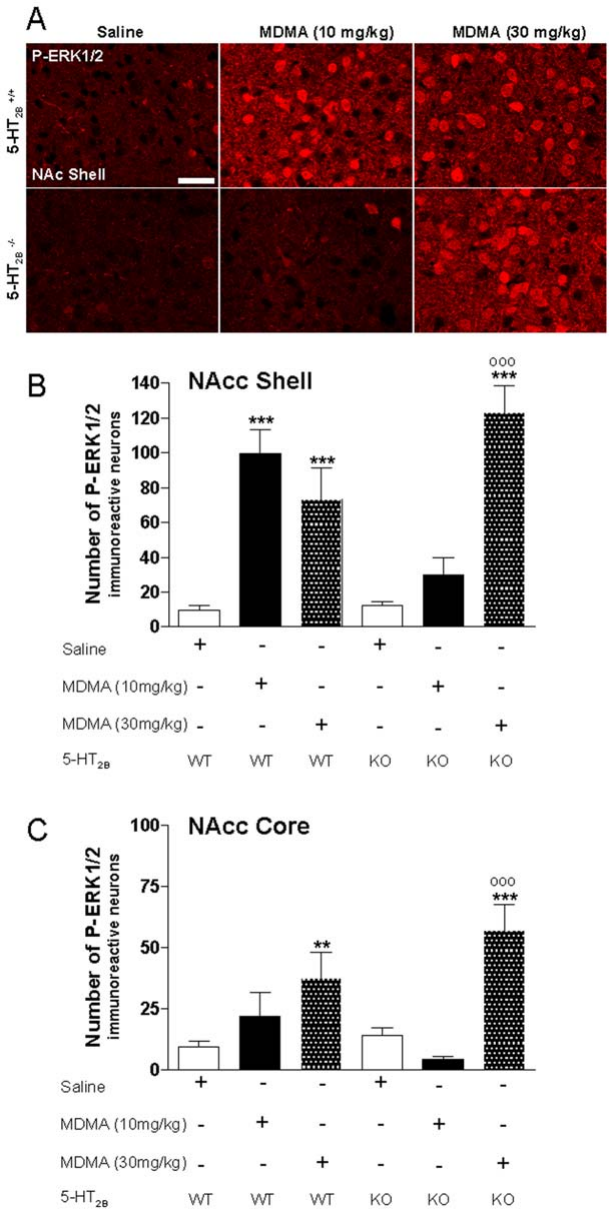
Effect of 5-HT_2B_ receptor on MDMA-induced ERK activation. 24 hours after CPP training (Figure 3), the same WT and 5-HT_2B_^−/−^ mice were re-exposed to saline, MDMA 10 mg/kg or MDMA 30 mg/kg before being processed for immunohistochemistry. **A**) Single confocal sections showing p-ERK1/2 immunoreactivity in the NAcc Shell of WT and 5-HT_2B_^−/−^mice 10 min after re-exposure. Scale bar: 40 µm. **B, C**) Quantification of p-ERK1/2 immunoreactive neurons in the NAcc Shell (**B**) and NAcc Core (**C**) of WT and 5-HT_2B_^−/−^ mice 10 min after re-exposure. Note the strength of ERK activation induced by re-exposure to both MDMA doses in WT animals. This activation was absent in 5-HT_2B_^−/−^ mice at 10 mg/kg MDMA, although it fully recovered at 30 mg/kg MDMA. Data (means±SEM; n = 4−10 mice per group) were analyzed using two-way ANOVA with genotype and treatment as main factors, revealing a significant interaction in NAcc Shell *F*(2, 33) = 21.27, p<0,001 and in NAcc Core, *F*(2, 33) = 4.77, p<0.05. A main effect of treatment was observed in NAcc Shell *F*(2, 33) = 45.29, p<0,001 and in NAcc Core, *F*(2, 33) = 21.85, p<0,001, but no effect of genotype neither in NAcc Shell *F*(1, 33) = 0.53, ns nor in NAcc Core, *F*(1, 33) = 0.21, ns. Bonferroni tests were used for post-hoc comparisons. The null hypothesis was rejected at the p<0.05 level; ** p<0.01, *** p<0.001 compared to saline-treated mice; °°° p<0.001, compared to MDMA 10 mg/kg treated mice.

Accumbal ERK stimulation participates in the long-lasting behavioral effects of drugs of abuse, [30] including CPP induced by MDMA [31]. This regulation required combined activation of dopamine and glutamate receptors. Thus, phosphorylation of ERK provides an index of post-synaptic DA receptor activation in the NAcc. The absence of DA release in the NAcc after acute MDMA (10 mg/kg) injection [19] and CPP after MDMA (10 mg/kg) conditioning indicates that mice lacking functional 5-HT_2B_ receptor should not exhibit ERK activation with low dose of MDMA. By contrast, DA release in the NAcc after acute MDMA (30 mg/kg) injection (Fig. 2) and CPP after MDMA (30 mg/kg) conditioning (Fig. 3) indicates that mice lacking functional 5-HT_2B_ receptor should exhibit ERK activation with high dose of MDMA. In order to assess this question, we quantified phosphorylated-ERK (p-ERK) immunoreactive neurons in the NAcc of WT and 5-HT_2B_^−/−^ mice 24 hours after the last MDMA (10 or 30 mg/kg) conditioning in the CPP paradigm (Figure 4A). Basal ERK phosphorylation was determined in WT or 5-HT_2B_^−/−^ mice that received saline injection during the CPP paradigm. As shown in Figure 4B, MDMA (10 mg/kg) re-exposure induced a 10-fold increase in p-ERK immuno-positive neurons in the NAcc (only in the shell) of WT mice expressing CPP. Conversely, in 5-HT_2B_^−/−^ mice, which did not express CPP after MDMA (10 mg/kg) conditioning, no significant increase of p-ERK immuno-positive neurons in the NAcc was seen after MDMA injection. However, a high dose of MDMA (30 mg/kg) induced—as it did in wild type mice— a 12-fold increase in p-ERK immuno-positive neurons in the NAcc (shell and core) of 5-HT_2B_^−/−^ mice expressing CPP compared to saline-injected 5-HT_2B_^−/−^ mice (Figure 4B and 4C).

### MDMA-Induced ERK Activation Occurs Only in D1R-Expressing NAcc Neurons

**Figure 5:**
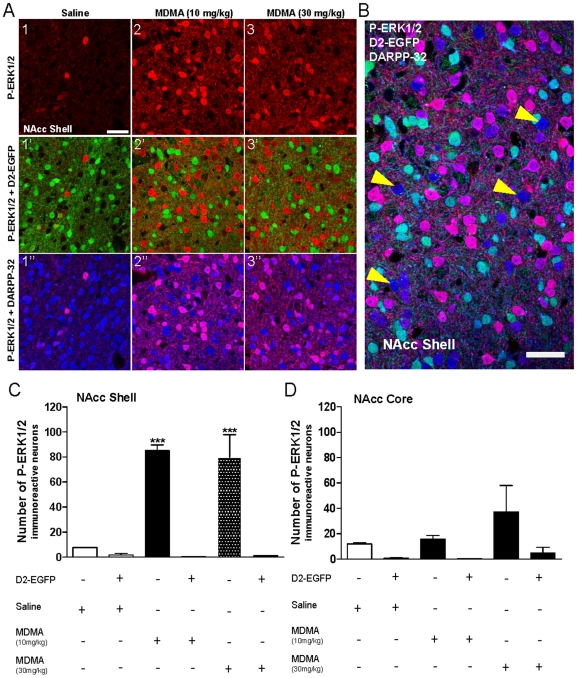
MDMA reexposure after CPP induces strong ERK1/2 activation exclusively in striatonigral medium-sized spiny neurons (MSNs) of the NAcc shell. *drd2*-EGFP BAC transgenic mice, in which EGFP expression is driven by the D2-receptor promoter, were trained for the same CPP protocol as in Figure 3 and re-exposed 24 hours after CPP training to saline, MDMA 10 mg/kg or MDMA 30 mg/kg. **A**) Single confocal sections showing p-ERK1/2 immunoreactivity (1–3, red) colocalized with D2-mediated EGFP (1′–3′, green) and DARPP-32 immunoreactivity (1″–3″, blue) in the NAcc Shell of *drd2*-EGFP mice 10 minutes after re-exposure to saline (1–1″), MDMA 10 mg/kg (2–2″) or MDMA 30 mg/kg (3–3″). The absolute segregation from D2-EGFP neurons in 1′–3′ and the complete colocalization with a fraction of DARPP-32-positive neurons in 1″–3″ reveal the identity of neurons expressing ERK1/2 activation, which are D1 receptor-containing striatonigral MSNs. Scale bar: 40 µm. **B**) p-ERK1/2 (red) + D2-EGFP (green) + DARPP-32 (blue) colocalized confocal image of the NAcc shell of an animal challenged with 10 mg/kg MDMA. Arrows point to neurons labeled only in blue, which are the small fraction of striatonigral MSNs that do not show ERK activation. Scale bar: 40 µm. **C**) Quantification of p-ERK1/2 immunoreactive neurons among EGFP-negative (−) or EGFP-positive (+) neurons in the NAcc Shell of *drd2*-EGFP mice 10 min after saline, MDMA 10 mg/kg or MDMA 30 mg/kg injections. **D**) Quantification of p-ERK1/2 immunoreactive neurons among EGFP-negative (−) or EGFP-positive (+) neurons in the NAcc Core of *drd2*-EGFP mice 10 min after saline, MDMA 10 mg/kg or MDMA 30 mg/kg injections. Data (means±SEM; n = 3 mice per group) were analyzed using two-way ANOVA with genotype and treatment as main factors, revealing a significant interaction *F*(2, 12) = 16.8, p<0,001 and a main effect of treatment *F*(2, 12) = 15.93, p<0,001 as well as of genotype *F*(1, 12) = 83.71, p<0.001. Bonferroni tests were used for post-hoc comparisons. The null hypothesis was rejected at the p<0.05 level; *** p<0.001 compared to saline-treated mice.

In order to characterize the population(s) of p-ERK immuno-positive neurons seen after MDMA (10 or 30 mg/kg) injection, we subjected *drd2*-EGFP transgenic mice to the same CPP protocol described above that responded as WT mice (not shown), and then counterstained brain section with a DARPP-32 antibody, a marker of GABAergic medium-size spiny neurons (MSNs). As shown in Figure 5, ERK phosphorylation occurred exclusively in DARPP32-expressing neurons, whereas no staining was detected in D2R (EGFP)-expressing neurons (Figure 5A and 5C). Almost all D1R-expressing neurons displayed ERK activation after MDMA injection (Figure 5B).

## Discussion

Sensitization is thought to underlie important aspects of vulnerability to drug addiction [28] and conditioned place preference (CPP) is a robust model used to assess the addictive properties of drugs of abuse. We show here that a “low” dose of MDMA (10 mg/kg) induces locomotor sensitization and CPP only in wildtype but not in 5-HT_2B_^−/−^ mice. These findings are consistent with: 1) our microdialysis data, showing that either pharmacological inhibition or permanent ablation of 5-HT_2B_ receptors is sufficient to block entirely both 5-HT and DA release in the NAcc following acute MDMA (10 mg/kg) injection [19], 2) lack of accumbal ERK activation in 5-HT_2B_^−/−^ mice following MDMA (10 mg/kg) conditioning (Fig. 4A). Both behavioral sensitization and CPP following repeated injection of low doses (10 mg/kg) of MDMA are thus entirely 5-HT_2B_ receptor-dependent. These data establish that 5-HT_2B_ receptors are critical for reinforcing effects properties and establishment of long-term alterations of behavioral responses to repeated exposure to MDMA.

Several lines of evidence indicate the involvement of the ERK pathway in long-term effects of drugs of abuse [30]. ERK is activated in reward-associated brain areas (including NAcc) through combined stimulation of DA and glutamate receptors after acute or repeated treatment with psychostimulant drugs [32]. Indeed, a previous study showed that MDMA (9 mg/kg)-induced locomotion and CPP were blocked by selective inhibitors of ERK [31]. We show here that a low dose of MDMA (10 mg/kg) induces ERK activation in WT but not in 5-HT_2B_^−/−^ mice. This is consistent with the absence of CPP in these mice following injection of a low dose of MDMA. Moreover, we find more robust ERK activation in the NAcc shell compared to the core following MDMA injection. This is in line with previous studies showing that ERK activation parallels the DA release seen in the shell compared to the core following MDMA injection [33], supporting a critical involvement of DA release in the shell for the rewarding properties of MDMA. Thus, 5-HT_2B_ receptors are critical for the CPP and ERK phosphorylation observed in mice following repeated injection of a low dose of MDMA.

In order to characterize the neurons that display increased p-ERK after MDMA injection, we used a DARPP-32 antibody, a marker of GABAergic medium-size spiny neurons (MSNs), in *drd2*-EGFP transgenic mice subjected to the CPP paradigm. MSNs account for >95% of the striatal neurons in rodents [34]. MSNs projecting to the substantia nigra pars reticulata and medial globus pallidus express mostly DA D_1_ receptors, whereas MSNs projecting to the lateral globus pallidus express preferentially DA D_2_ receptors [35]. These two subpopulations are homogenously distributed throughout the striatum, and are known to have opposite effects on behavior [35], [36]. MDMA-induced locomotor activity, behavioral sensitization [37], [38], self-administration [39] and pERK stimulation [40] have all been shown to be reduced in D_1_ receptor antagonist-treated mice. We show here that ERK phosphorylation, following MDMA injection, occurs exclusively in DARPP32-expressing neurons, whereas no ERK labeling was detected in D_2_ receptor (EGFP)-expressing neurons. A recent study showed that in this transgenic mouse line, 100% of the MSNs express either D_1_ or D_2_ receptors [41]. Thus, our results demonstrate that the ERK activation induced by repeated MDMA injection occurs selectively in D1 receptor-striatonigral MSNs in the NAcc (shell and core). This complete segregation between the striatonigral and striatopallidal circuits within the striatum, in response to MDMA injection, was independently reported for cocaine [23]. Activated ERK, in turn, induces CREB phosphorylation, c-Fos expression, mitogen- and stress-activated kinase-1 (MSK1) activation and histone H3 phosphorylation, all of which further regulate gene expression in D_1_ receptor-expressing neurons [42], [43]. These gene expression changes may contribute to drug-induced persistent neuroadaptations (i.e., alterations of dendritic morphology, synaptic transmission and synaptic plasticity) [42]–[44]. Our work indicates that MDMA induces DA-dependent ERK activation in D_1_ receptor-expressing neurons that likely triggers the same signaling cascade as other drugs of abuse.

Our data show that unlike a “low” dose of MDMA (10 mg/kg), a high dose (30 mg/kg) induces locomotor sensitization and CPP in 5-HT_2B_^−/−^ mice. Since MDMA binds to DAT with a lower affinity than SERT [29] it may, at low (10 mg/kg) or high (30 mg/kg) doses, mostly bind to and reverse SERT or both SERT/DAT, respectively. The contrasting behavior observed between low and high doses in animals provides some evidence that the dose of MDMA affects its mode of action [45]. These findings are consistent with our microdialysis data, showing that either pharmacological inhibition or permanent ablation of 5-HT_2B_ receptors is sufficient to block entirely 5-HT and DA release in the NAcc following a “low” dose of MDMA [19] while a “high” dose of MDMA induces DA release without 5-HT release (fig. 2). These findings support the notion that, according to the dose, MDMA has different pharmacological targets leading to different behavioral effects. A low dose of MDMA induce a 5-HT_2B_ receptor-dependent 5-HT release that most likely promotes subsequent DA release in NAcc, while a “high” dose activates also a 5-HT_2B_ receptor-/5-HT-independent DA release, probably via a direct effect of MDMA on DAT. In this regard, a recent study showed that, according to the dose, MDMA has a dual effect (5-HT dependent or independent) on DA neurons firing [46]. Although some literature supports these observations (i.e., the released 5-HT drives the release of DA), other data showed that fenfluramine, a selective 5-HT releaser, fails to induce DA release [47]. This is probably due to the differential 5-HT releasing capacity of fenfluramine compared to MDMA [48] and the complexity of the regulation of mesolimbic DA activity by 5-HT and its receptors (i.e. differences in the affinities of the various 5-HT receptors for 5-HT, differential effects of 5-HT agonists on second-messenger systems, different receptor desensitization and the nature of the 5-HT receptor expressing neurons) {See [49] and [4] for an extensive discussion}. Alternatively, MDMA and its N-demethylated metabolite 3,4-methylenedioxyamphetamine (MDA) each preferentially bind to and activate human recombinant 5-HT_2B_ receptors at concentrations close to those reported in plasma after a single recreational dose. MDMA and MDA, elicit also a prolonged mitogenic responses in human valvular interstitial cells, via direct activation of 5-HT_2B_ receptors [50]. Thus, the behavioral effect of MDMA may be mediated, in part, through direct action on postsynaptic 5-HT_2B_ receptors.

That a high dose of MDMA-induced CPP in 5-HT_2B_^−/−^ mice in a DA-dependent fashion (accumbal ERK activation) suggests that DA release in NAcc is sufficient for the rewarding properties of MDMA whatever mode of DA release (i.e with or without a supportive role of 5-HT). Moreover, the lack of 5-HT release in 5-HT_2B_^−/−^ or RS127445-treated mice following acute injection of MDMA (30 mg/kg) suggest that MDMA induces CPP and stimulates ERK phosphorylation through an entire 5-HT-independent but DA-dependent mechanism.

The mean recreational dose of MDMA in humans (oral route) is about 2 mg/kg (70–200 mg of MDMA per pill) [51]. In line with the principle of interspecies drug dose scaling–also still a matter of debate– the low dose of MDMA used in mice is most likely a “high” human dose [52]. This supports the notion that pharmacological blockade of the 5-HT_2B_ receptors in Human might counteract the effect of MDMA after a recreational dose as it did in mice. Unlike SSRIs, the 5-HT_2B_ receptor antagonist RS127445 completely blocks MDMA induced 5-HT and DA release, in response to low dose of MDMA. Moreover, RS127445 has no effect on basal 5-HT extracellular concentration, unlike SSRIs which may induce serotonin syndrome due to a synergistic increase in synaptic serotonin [53]. The fact that reinstatement after CPP extinction is abolished by RS127445, a selective 5-HT_2B_ receptor antagonist in WT mice highlights the putative clinical efficacy of 5-HT_2B_ receptor blockade in the treatment of MDMA abuse. Antagonists of 5-HT_2B_ receptor could serve as promising therapeutic drugs for the prevention of the acute and long-term effects associated with MDMA use, and could be a way to avoid relapse in abstaining MDMA users. In line with this hypothesis, a series of atypical antipsychotics (clozapine, amisulpride, metoclopramine, olanzapine and aripiprazole) that have potent 5-HT_2B_ receptor antagonist properties would be expected to be effective prophylactics against MDMA abuse [54]–[56]. Indeed, some antipsychotics reduces MDMA-stimulated locomotion and hyperthermia, effects believed to be mediated at least in part by the 5-HT_2A_ receptor antagonist properties [6], [57], [58]. The contribution of 5-HT_2B_ receptors to the atypical profile of some benzamide antipsychotic has yet to be investigated, in respect to psychostimulants addictive effects.
